# Persistent Organic Pollutants in Human Milk from Central Italy: Levels and Time Trends

**DOI:** 10.5402/2011/107514

**Published:** 2011-07-17

**Authors:** Cristiana Guerranti, Michela Palmieri, Michela Mariottini, Silvano Ettore Focardi

**Affiliations:** Department of Environmental Sciences, University of Siena, Via P.A. Mattioli, 53100 Siena, Italy

## Abstract

Persistent organic pollutants (POPs) such as HCB, *p,p*′-DDE, and PCBs were measured in Italian breast milk. This work is part of a study on human milk, adipose tissues, and food carried out in the same area over the last 20 years. The results showed the prevalence of *p,p*′-DDE and PCBs over HCB. Comparison of our results with those of previous studies carried out in the same area showed that concentrations are decreasing. No statistically significant differences in organochlorine levels were found when the samples were divided into maternal age classes and into the categories “primiparae” and “multiparae”. In order to quantify the amount of the molecules of interest transmitted by mother to child during breast feeding, we estimated the daily intake of each class of compounds: our results indicated that HCB and *p,p*′-DDE were several times lower than the safety thresholds.

## 1. Introduction

The presence of organic pollutants in a mother's milk was first reported in 1951 by Laug et al. [1], who analyzed 32 samples of milk from women in Washington D.C. (USA) for the presence of the insecticide DDT and its metabolites. Jensen [2] subsequently reported the presence of PCBs in mother's milk (in 1966), and many other chemical contaminants have been detected in breast milk since then. Analyses of breast milk for environmental chemicals are of broad scientific interest, since human milk can be used as an indicator for the general level of contamination in a population, and thus it is useful to assess potential health risks associated with the intake of human milk. Large quantities of some chemicals may be transferred from mother to child through human milk [3]. In a study carried out in the Netherlands in 1999, it was estimated that breast feeding for 6 months accounts for between 12 and 14% of exposure to some organochlorinated compounds in the first 25 years of life [4]. Various factors can increase the level of contamination in breast milk, including the mother's age (higher age is associated with a greater body burden of contaminants), the number of children (primiparae excrete more contaminants in their breast milk), the duration of previous breast feeding, and distance in time since this (in multiparae); other events that may have mobilized maternal fat deposits (such as weight loss treatments) and diet (in particular fish consumption) [5]. If we consider age as a predictive factor of plasma of PCB concentration, as age is correlated with the years of exposure to contaminated foods, we should also mention that although the presence of such molecules in food has decreased over the years, the half life of high chlorinated PCBs can vary from eight to over seventeen years [6]. In this study, the presence and levels of some classes of organochlorinated compounds was evaluated in samples of breast milk. In order to quantify the consumption by infants of the contaminants studied, their intake through breast milk was estimated. In fact, in many cases, human milk is the only food that newborns consume in the first months of life, such an analysis, therefore, makes it possible to estimate the intake of xenobiotics in a more simple and accurate manner than can be done in organisms with a more complex diet. This work is part of a long series of studies on human exposure to environmental contaminants in the Siena area. Organochlorines have been studied in human adipose tissue samples [7–11] and human milk [12]; organohalogen compounds have been determined in food and diet [13–15], and perfluorinated compounds have been identified in human blood and serum [16].

The data acquired in this study have been compared with previous studies [12], in order to determine the temporal trend of contaminant concentrations in the breast milk of women residing in the study area. The results reported in this paper, together with those published previously [14] regarding the polybrominated diphenyl ethers (PBDEs) concentrations in the same samples, are intended to provide a detailed picture of contamination in breast milk in central Italy.

## 2. Materials and Methods

### 2.1. Sample Collection

Individual breast milk samples were obtained from women living in the Siena area, following the ethical standards and the approval of the Local Ethics Committee and the acquisition of informed consent. A total of 50 milk samples were collected about 8–10 days after delivery.

Information such as age, number of births, occupation, smoking, dietary habits, and so forth was recorded for all mothers. Participants in the study were on average 30.8 ± 4.1 years of age and had lived in the area for at least 5 years. The human milk (about 50 mL) was manually expressed into 100 mL contamination-free pots, and appropriate precautions (such as previous rinse with hexane) were taken to prevent the contamination of the samples. All samples were kept frozen at −20°C until analysis.

### 2.2. Sample Analysis

OC pesticides and PCBs were extracted according to a wide tested method [15]. Milk samples (10–12 g) were homogenized with sodium sulphate and soxhlet extracted with methylene chloride/hexane (3 : 1 v/v) for 16 hours. After extraction, a portion of each extract was used for gravimetric determination of the total lipid content of the milk. The extract was rotary evaporated at 34°C. A multilayer silica gel column was prepared by packing a glass column (20 mm internal diameter, i.d.) with a series of silica gel layers. The column was cleaned with 150 mL of hexane, then the samples were eluted with 200 mL of hexane and reduced to 1 mL using hexane. OC pesticides and PCBs were analyzed by gas chromatography using a Perkin Elmer Autosystem gas chromatograph fitted with a 63Ni electron capture detector (GC-ECD). The capillary column (SBP-5, Supelco) was 30 m long and 0.25 mm i.d., with a 0.25 *μ*m thick coating. Helium was used as the carrier gas. Injection was set at splitless mode, the injector temperature was 270°C, and the column was programmed to be heated from 120°C to 280°C at 5°C/min, then maintained at 280°C for 10 minutes. The detector temperature was 300°C, and the makeup gas was argon/methane (95/5). A mixture of the pesticide HCB, *p,p′*-DDE (the main DDT's metabolite) (Dr. Ehrenstorfer GmbH), Aroclor 1260 and 1254 (Supelco), and mono-*ortho *PCBs (Dr. Ehrenstorfer GmbH) were used as external standards and for calibration. Sample results were confirmed with a GCQ plus ion trap mass spectrometer (MS) from ThermoFinnigan in selected ion-monitoring mode (SIM). A trace gas chromatograph was equipped with an AS 2000 autosampler (ThermoFinnigan) and fitted with an Rtx-5MS capillary column (30 m × 0.25 mm i.d., 0.25 *μ*m) from Restek. GC conditions and information on target/qualifier ions are described elsewhere [17]. The concentrations of individually resolved peaks were summed to obtain total PCB concentrations, calculated on lipid weight (l.w.). 

The procedures described above were checked for recoveries and reproducibility. Quality assurance for the measurement of OC pesticides and PCBs and for the technique was confirmed by analyzing certified reference materials provided by the *Community Bureau of Reference *(BCR) of the Commission of the European Communities, specifically, natural milk powder no. 187 for pesticides and no. 450 for PCBs. The results of two replicates were highly consistent with the certified values (mean errors: 4% for pesticides and 5% for PCBs). A blank prepared according to the same procedure used for the samples was included every five samples, and the results were blank corrected. The limit of detection (LOD), calculated as mean blank + 3SD, was 0.01 ng/g lipid for OC pesticides and PCBs. PCB-30 and PCB-209 were added to each sample as recovery standards and recoveries were ever higher than 88%.

### 2.3. Statistical Analysis

**Figure 1:**
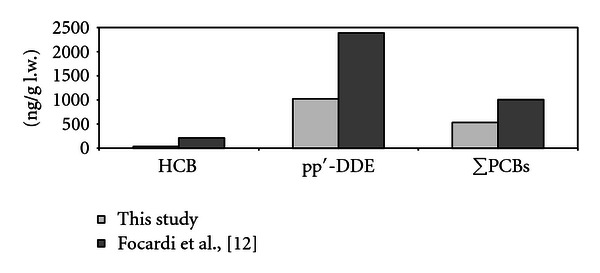
Time trend of OC concentrations (mean levels in ng/g l.w.) in human breast milk from central Italy.

**Figure 2:**
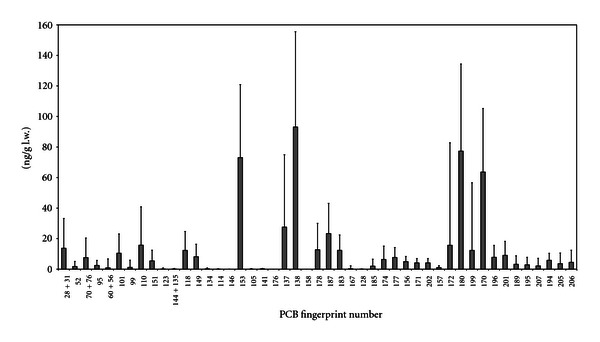
PCB Fingerprint (mean value ± SD in ng/g l.w.) in breast milk samples.

Statistical analysis was performed using the Kruskal-Wallis one-way ANOVA by ranks, the Wilcoxon-Mann-Whitney test, and Spearman's correlation coefficient (rs). A probability level of less than 0.05 was considered significant. Statistical analysis, as well as the graph reported in Figures 1 and 2, was made using the programmes Microsoft Excel 2003 (Microsoft Corporation) and Statistica 7.1 (Statsoft Inc.), both for Windows XP.

## 3. Results

Fifty milk samples were analyzed by GC-ECD, and the results are shown in Table 1. *p,p′*-DDE and PCBs were detected in almost all samples (95% and 88% of samples, resp.), whereas just few samples (about 30%) showed HCB in detectable concentrations. Of the OC pollutants, *p,p′-*DDE prevailed, followed by PCBs and HCB. OC concentrations reported in this study were lower than those measured in milk samples collected previously from the same geographic area (the extraction method used was similar to that above described, and concentrations were determined by GC-ECD). Focardi et al. [12] reported mean concentrations of *p,p′*-DDE, HCB, and PCBs in human milk of 2387, 214, and 1007 ng/g l.w., respectively (Figure 1).

Fifty-one congeners were singled out from the PCB residue (Figure 2). The most abundant congeners included PCB-138, PCB-153, PCB-180, PCB-170, and PCB-187. The order of prevalence of the congeners was PCB-153>PCB-180>PCB-138>PCB-170>PCB-118>PCB-187. Heptachlorobiphenyls accounted for 35% of the total, followed by 33% for hexachlorobiphenyls and by 19% for pentachlorobiphenyls.

The samples analyzed were divided into age classes and according to the mother's number of deliveries (Table 2). The mean values of *p,p′*-DDE, HCB, and ΣPCB were higher in the 35–40-year age class in relation to those of other classes, while there were no statistically significant differences in the organochlorinated compound concentrations between the categories “primiparae” and “multiparae”. 

All study participants reported low consumption of fish products, and the large majority declared to be no smoker; levels of OCs were not significantly associated with maternal age, potential occupational exposure, and particular dietary habits.

### 3.1. Estimated Contaminant Intake through Breast Milk Consumption

The data obtained from these analyses permitted us to estimate the quantities of the OCs studied that breastfeeding infants ingested through their diet. Considering an average daily intake of approximately 150 g of milk, a mean figure referring to the first week of life of a newborn baby [18], the daily intake was found to be 0.36 *μ*g of HCB, 8.61 *μ*g of *p,p′*-DDE, and 5.38 *μ*g of PCBs.

## 4. Discussion

Apart from the excretion of OC pollutants through bile and urine, milk represents an alternative route of elimination in lactating animals, including humans. Variations in OC concentrations might be expected to occur in relation to factors such as the efficiency of absorption and excretion, the mother's age, and her nutritional and socioeconomic status. The prevalence of *p,p′-*DDE over PCBs and HCB has previously been reported [11] in the adipose tissue of women living in Siena; the same study confirms also that HCB can be found in a limited fraction of the samples analyzed, probably due to a low use of this pesticide in the study area. HCB, *p,p′*-DDE, and PCB levels were similar to concentrations detected in breast milk in the U.K. in the years 2001–2003 (range: ND-180; 22–1600; 26–530 ng/g l.w., resp.) [19], and in breast milk from Russia [20] in the year 2000 (*p,p′*-DDE 943 ng/g l.w and HCB 42.2 ng/g l.w.). Concerning PCBs, the results of this study are higher than those reported in Norwegian breast milk samples [21] collected in the years 2000-2001 (mean values 159–278 ng/g l.w.), in samples from Russia [20] in 2003 (median 283 ng/g l.w.), and for samples of breast milk from two large Chinese cities [22] (mean values 31 ng/g l.w. and 35 ng/g l.w.). In the latter study, however, the mean DDE values were twice as high as those reported in our data (2,480–2,850 ng/g l.w.). 

Comparing our data with those previously obtained for the same area [12], a noticeable decline in OC contamination can be observed, with mean values resulting approximately 40% lower. Reduced contamination levels in human tissues in the years following antipollutant regulations have also been observed in other countries [22]. Kalantzi et al. [19] showed a decreasing trend in OC levels detected in breast milk (U.K.) since the sixties, probably as a consequence of bans imposed on the use of these contaminants. Dewailly et al. [23] described statistically significant decreases in HCB, DDT, and PCBs in human adipose tissue from Canada in the period 1969 to 1985. Loganathan et al. [24] analyzed samples collected in Japan from 1928–1985, finding that the decline over the years seemed to correspond to the regulatory controls, especially regarding DDT levels.

PCB-138, PCB-153, PCB-180, PCB-170, PCB-137, and PCB-187 prevailed: these are the most common congeners in commercial mixtures and the environment [25]. The previous studies carried out in the Siena area [8–11, 13] confirm that the same group of congeners are the most common in human tissues and food; the difference from past studies is the prevalence in our samples of milk of PCB-138 rather than PCB-153. In fact, PCB-153 is the most common congener, due to its high persistence and low environmental degradability, as well as its characteristic of being difficult for organisms to metabolize [26, 27].

Hexa-CBs and hepta-CBs prevailed in Italian milk samples. They are the most common groups of isomers, partly because they have been widely used in commercial mixtures, such as Aroclor 1260 [28]. Concentrations of low chlorinated PCBs (tetra-CBs) in the fingerprints were much lower than those of other congeners. This finding may be linked to the fact that these PCBs are more easily metabolized and are less common in commercial mixtures. 

The PCB congener prevalence obtained in this work are in accordance with those of other studies on breast milk [23, 29–31].

Age and exposure to persistent contaminants usually generate a positive correlation [32, 33], therefore, the lower the average age, the less the individual is likely to be exposed to contaminants. No significant correlation was found in this study between age and POP concentrations, neither were the differences between age classes statistically significant, even though the 35–40 age class showed the highest concentrations of OC compounds on average (Table 2).

Similarly, the study carried out on milk samples from Russia [34] also found no statistically significant difference in OC levels between primiparae and multiparae (Table 2), although the milk of primiparae should have higher OC concentrations than that of multiparae, as OCs accumulate throughout the years before pregnancy.

The FAO-WHO have defined acceptable intakes (known as tolerable daily intakes (TDI)) for a series of environmental contaminants. No TDI has been established for HCB, but an acceptable daily intake (ADI) of 0.6 *μ*g/kg body weight (b.w.) is considered below any safe exposure threshold [35]. For compounds in the DDT group, an initial assessment established an ADI of 20 *μ*g/kg b.w. [36], which was subsequently updated to a provisional TDI (PTDI) of 10 *μ*g/kg b.w. [37]. There is no ADI for PCBs, nor a TDI specifically for them that is, for PCBs considered distinctly from dioxin and furan levels.

Considering a mean weight of breastfeeding babies of 3.5 kg [38, 39], the calculated intake values are below the safety thresholds of 17% for HCB and 24.6% for *p,p′*-DDE; therefore, the quantities of these OC pesticides consumed by breastfeeding babies in the Siena area are below the safety threshold for the health of infants whose diet consists exclusively of their mother's milk.

## 5. Conclusions

*p,p′*-DDE and PCBs were detected in varying proportions in all samples, possibly due to the efficiency of absorption and excretion, and the mothers' age, nutritional, and socioeconomic status. Of the OC pollutants, *p,p′-*DDE prevailed, followed by PCBs and HCB. The HCB, *p,p′*-DDE, and PCB levels were similar to concentrations reported in milk samples from other parts of Europe. It emerged from a comparison with a previous study carried out on breast milk samples from the same area [12] that the presence of OCs has decreased between the 1980s and the present day, thus, confirming a European decline, corresponding to the regulatory controls applied. PCB-138 prevailed over PCB-153, in contrast to previous studies in the same area, while hexa-CBs and hepta-CBs were predominant, the latter have both been widely used in commercial mixtures, such as Aroclor 1260.

Age and exposure to persistent contaminants usually generate a positive correlation; the division of the samples into maternal age classes revealed that the class with the highest concentration was that of 35–40-year old women, although the difference was not statistically significant. Dividing the milk samples into two groups—from primiparae and from multiparae—did not reveal any clear differences in values between the two groups.

Exposure to xenobiotics can represent an important factor in determining increasingly common problems in children, such as immunological, neurological and behavioral, or developmental disorders. Moreover, epidemiological studies have suggested a relationship between cancer and exposure to POPs, some of which act as carcinogens [40]. However, it must also be emphasized that breastfeeding appears to have a protective effect against breast cancer in the mother [18]. Despite the presence of contaminants in the breast milk studied, the estimated intakes of OC pesticides were below safe threshold levels. These results and the clear decreasing trend that emerged from the comparison with a previous study carried out in the same area support breastfeeding, as there is a lack of evidence of risks due to the presence of contaminants in milk from this area, especially considering the psychological and nutritional importance of this practice. Nevertheless, preventive measures, such as food monitoring and dietary intake assessment, need to be adopted in order to reduce the body burden of OC pollutants and to ensure that the decrease in OC levels that began in the nineties can continue.

## Figures and Tables

**Table 1:** HCB*, p,p′*-DDE and PCB mean and median concentration (ng/g l.w.) from Italian breast milk (*n *= 50).

ng/g b.l.	Mean (S.D.)	Median
HCB	38.66 (46.40)	21.09
*p,p′*-DDE	1024 (808.44)	867.86
*∑*PCB	532.87 (309.79)	562.24

**Table 2:** Mean (S.D.) and median concentrations (ng/g l.w.) of HCB, *p,p′*-DDE and PCB in Italian breast milk samples divided into age classes and according to number of births.

ng/g l.w.		Maternal age (years)				Births	
		20–25	25–30	30–35	35–40	Primiparae	Multiparae
		*n *= 7	*n *= 17	*n *= 19	*n *= 7	*n *= 37	*n *= 13

HCB	Mean	36.10 (38.52)	38.54 (44.31)	21.97 (21.20)	70.63 (98.29)	40.62 (51.52)	24.37 (20.37)
	Median	22.17	14.79	14.37	32.78	17.82	18.12

*p,p′*-DDE	Mean	870.80 (815.28)	717.08 (402.17)	1107.74 (676.15)	1548.45 (1448.78)	957.77 (774.08)	1149.69 (836.40)
	Median	468.87	639.29	1098.49	1341.02	853.02	1341.02

*∑*PCB	Mean	535.13 (239.88)	490.84 (316.96)	573.16 (374.78)	573.46 (418.84)	582.46 (299.04)	541.66 (308.63)
	Median	628.37	416.30	585.37	591.30	601.76	585.37

## Abbreviations

DDT: dichlorodiphenyltrichloroethane
HCB: hexachlorobenzene
OCs: organochlorines
PBDEs: polybrominated diphenyl ethers
PCBs: polychlorinated biphenyls
*p,p′*-DDE: dichlorodiphenyldichloroethylene.
